# Recent Advances in Bioelectrochemical Systems for Nitrogen and Phosphorus Recovery Using Membranes

**DOI:** 10.3390/membranes13020186

**Published:** 2023-02-02

**Authors:** Míriam Cerrillo, Victor Riau, August Bonmatí

**Affiliations:** Sustainability in Biosystems, Institute of Agrifood Research and Technology (IRTA), Torre Marimon, Caldes de Montbui, E-08140 Barcelona, Spain

**Keywords:** ammonium, phosphorus, bioelectrochemical systems, membrane, migration

## Abstract

Bioelectrochemical systems (BESs) have emerged as a technology that is able to recover resources from different kinds of substrates, especially wastewater. Nutrient recovery, mostly based on membrane reactor configuration, is a clear niche for BES application. The recovery of nitrogen or phosphorus allows for treatment of wastewater while simultaneously collecting a concentrated stream with nutrients that can be reintroduced into the system, becoming a circular economy solution. The aim of this study is to review recent advances in membrane-based BESs for nitrogen and phosphorus recovery and compare the recovery efficiencies and energy requirements of each system. Finally, there is a discussion of the main issues that arise from using membrane-based BESs. The results presented in this review show that it would be beneficial to intensify research on BESs to improve recovery efficiencies at the lowest construction cost in order to take the final step towards scaling up and commercialising this technology.

## 1. Introduction

Bioelectrochemical systems (BESs) are bioreactors that are equipped with two electrodes, an anode, and a cathode and use exoelectrogenic microorganisms to catalyse oxidation and/or reduction reactions. BESs have become a highly versatile technology that allows wastewater treatment to be combined with the recovery or production of compounds such as nutrients and energy carriers. BESs can be operated as a stand-alone technology or in combination with other technologies, such as anaerobic digestion, in order to improve the recovery of resources and energy within a circular economy approach [1,2,3,4].

BESs can be used to treat a wide range of organic substrates and wastewaters, such as digested sludge and agroindustry, food industry, and urban wastewater [5]. In a changing paradigm in waste and wastewater management technologies from the purpose of waste disposal to the biorefinery concept, the recovery and reuse of nutrients from wastewater is a priority over their disposal through technologies such as the nitrification–denitrification process [6]. For example, fertilisation activities are highly dependent on ammonia and phosphate, and their recovery from waste streams will reduce the demand for phosphate mined from rock or ammonium produced by industrial processes. Ammonium is industrially produced by nitrogen conversion from atmospheric N_2_ gas through the Haber–Bosch process, which is energy intensive due to the need to supply high temperatures (400–500 °C) and high pressure (100–200 atm) to the reactors. Furthermore, 1.87 tonnes of carbon dioxide (CO_2_) is generated with every tonne of NH_3_ produced by the Haber–Bosch process, contributing 1.2% of global anthropogenic CO_2_ emissions [7]. With regard to phosphate, most of it currently comes from a non-renewable resource, phosphate rock, which has no feasible substitute and can be contaminated by heavy metals. Mining costs increase over time as P rock becomes less available, constraining further mining action. Without proper management, phosphate rock is projected to be depleted in the next 70–140 years [8].

Although BESs can be configured as a single-chambered cell, the most typical setting is a dual-chambered device consisting of an anodic compartment and a cathodic compartment separated by a membrane. This configuration has been widely investigated for nutrient recovery, with different kinds of membranes tested to increase the selectivity of the recovered compound. The most commonly used membranes in the construction of BESs are ion-exchange membranes (IEM), typically to recover ammonium and phosphates for reuse as fertilisers.

BESs have been the subject of various literature reviews focused on materials and their many applications [9,10] along with nutrient removal and recovery [11,12,13], including membrane and membraneless reactors. The most recent reviews focused specifically on the recovery of nitrogen from a sustainability perspective [14,15]. To the authors’ knowledge, no reviews concerning ammonia and phosphate recovery using membrane-based BESs have to date been published. Furthermore, in the last few years, several papers have dealt with nitrogen and phosphate recovery including new advances and focusing both on achieving high efficiency in nutrient recovery and reducing the associated chemical requirements and energy consumption. Thus, an update on new advances in this field is needed.

The main objectives of this review are to highlight the latest advances in nitrogen and phosphorus recovery using different kinds of membrane-BES configurations and compare the recovery efficiency and energy requirements of each system. In addition, the upscaling of BESs, which is gradually being carried out, is reviewed.

## 2. Methodology

A literature review was undertaken based on a bibliometric analysis of publications in the field of nutrient recovery using membrane-based BESs. The source used to identify these publications was the Web of Science database. In order to include the most recent advances in this field, the research was performed on studies published between 2016 and 2022. The following main keywords were used for the selection: nutrient, ammonia, ammonium, phosphorus, phosphate, bioelectrochemical systems, microbial fuel cell, and microbial electrolysis cell. The obtained papers were analysed to choose only those related to membrane-based reactors. As a result, 49 papers were selected for inclusion in this review.

## 3. Principles of Nutrient Recovery in BESs Using Membranes

BESs for nutrient recovery generally consist of an anodic compartment and a cathodic compartment divided by a membrane. Exoelectrogenic bacteria grow on the anode, forming a biofilm, and are capable of oxidising organic matter that is introduced to the anode compartment. The electrons and protons produced by the organic matter oxidation at the anode are directed to the cathode compartment by an external electrical circuit and through the internal membrane, respectively, and are combined by a reduction reaction. The circulation of electrons through the external electrical circuit generates electricity (microbial fuel cell, MFC). A charge imbalance is produced in the cell due to electron transfer from the anode to the cathode, which leads to positive charge migration, such as of protons, through the cation-exchange membrane (CEM, the most common IEM for ammonia recovery). However, when complex substrates are introduced to the anode compartment, other cations such as NH_4_^+^ will be in higher concentrations than the protons and preferably transfer (migrate) to the cathode compartment against the protons. In a similar way to ammonium, when an anion-exchange membrane (AEM) is used as a separator, phosphate ions can migrate from the cathode to the anode compartment.

Since cation/anion migration through the CEM or AEM is linked to the amount of charge circulating through the external electrical circuit, the transfer rate can be improved by an external power source connection in addition to producing hydrogen gas or other nonspontaneous reactions in the cathode (microbial electrolysis cell, MEC). Ammonia recovery improves with higher current densities; therefore, externally powered MECs perform better than MFCs for the recovery of ions. On the other hand, in the cathode chamber of CEM-equipped MFCs and MECs, a local high pH is created around the cathode surface through proton consumption or release of hydroxyls, which may improve nutrient recovery, i.e., by inducing ammonium conversion to ammonia or struvite crystallisation [16], as discussed in Section 3.1 and Section 3.2.

**Figure 1:**
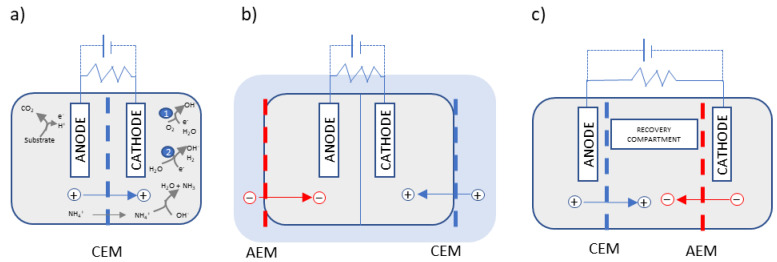
Examples of three typical configurations of membrane-based BESs. + and − stand for ions charged positively (i.e., NH_4_^+^) and negatively (i.e., PO_4_^3−^), respectively. (**a**) Dual-chambered BESs, (**b**) submersed BESs [22], and (**c**) three-chambered cell with recovery compartment. The main reactions taking place in BESs are shown in (**a**) by way of example, where ❶ and ❷ stand for MFC and MEC cathodic reactions, respectively.

Membranes are a key element in nutrient recovery in BESs since they allow for the separation of nitrogen and phosphorus from the wastewater matrix and subsequent recovery in a clean solution, in contrast to BESs without a membrane. Several types of IEMs have been applied in the construction of BESs, such as proton-exchange membranes (PEM), CEMs, AEMs, ultrafiltration, microfiltration, and bipolar membranes. Comprehensive reviews of membrane separators in BESs have been published in recent years [17,18]. Membrane-BESs can be configured in different setups using a unique membrane or a combination of different kinds of membranes, achieving different ways of recovering nutrients, as shown in Figure 1.

Although membranes are of great importance in the recovery of nutrients in BESs, reactor performance has been reported to be significantly affected by IEMs since they generally increase the internal electrical resistance and coulombic efficiencies, but also in relation to capital cost, as they represent up to 40% of the total cost of BESs [19]. Nevertheless, membrane-based BESs for nutrient recovery are a subject of great interest, as demonstrated by the 49 papers published since 2016 included in this review related to ammonia recovery (63%) and combined ammonia and phosphate recovery (37%). Although phosphate recovery or remobilisation from iron phosphate in BESs has been reported [20,21], combined nitrogen and phosphorus recovery systems are usually developed.

### 3.1. Ammonia Recovery by Stripping and Absorption

In CEM-equipped BESs, NH_4_^+^ migrates to the cathode chamber through the membrane combined with other cations (Figure 1a). In order to obtain a pure ammonia solution, a subsequent step of stripping and absorption is usually applied. The stripping and absorption process consists of injecting an air flux through the catholyte to drag dissolved gases, in this case ammonia gas. The gaseous stream then comes into contact with an acidic solution, where the ammonia is absorbed, transforming it again into ammonium. In conventional ammonia stripping, the temperature or pH of the substrate needs to be externally increased to favour a shift in the ammonium/ammonia equilibrium towards the second gaseous species. While in BESs, this step is greatly enhanced with no need for an alkali addition or temperature increase, thus reducing reactant costs and energy consumption due to the catholyte spontaneous increase in pH value [14]. Using a NaCl solution in the cathode compartment, the catholyte pH value has been reported to increase from 9.1 to around 10.8–12.1 at the end of the batch [23].

### 3.2. Nutrient Recovery by Precipitation

Combined nitrogen and phosphorus recovery can generally be achieved by obtaining a concentrate solution or by struvite precipitation. Struvite is a white salt that crystallises with an orthorhombic structure under alkaline conditions when magnesium, ammonium, and phosphate are present in equimolar concentrations, as shown by reaction (1). Struvite is considered to be a slow-release fertiliser.

Mg^2+^ + NH_4_^+^ + H_n_PO_4_^3−n^ + 6H_2_O → MgNH_4_PO_4_ + 6H_2_O + nH^+^ (1)

The cost of struvite crystallisation, which in conventional reactors can be high due to pH adjustment using basic chemicals [24] such as Mg(OH)_2_, NaOH, and Ca(OH)_2_, can be reduced thanks to catholyte basification in BESs.

## 4. Nitrogen Recovery Using Membranes in BESs

Recovery of nitrogen in BESs through the use of membranes has been widely studied in recent years with different configurations. As described in Section 3, in most cases, ammonia recovery in BES is based on ammonium migration in combination with other cations through a CEM by two-chambered cells. Other setups have been developed to increase ammonia recovery efficiencies or decrease costs or electrical energy input. Table 1 presents a summary of the main data from the most recently published works. The specific ammonium recovery rate (g_N_ m^−2^_membrane_ d^−1^) is used in this paper, normalising data supplied from the different studies as required, in order to facilitate a comparison of performance identified in the various studies.

This section divides nitrogen recovery BESs into three different groups: the most typical two-chambered cells, the hydrophobic membrane-BES configuration, and multiple compartment cells.

### 4.1. Two-Chambered BESs Equipped with CEM for Ammonia Recovery

One of the possible applications of BESs with two compartments is ammonia recovery from high-strength wastewater (i.e., livestock manure). Great efforts have been made to optimise the operation. The cathode chamber of an MFC continuously fed with pig slurry was coupled to a stripping and absorption unit, achieving a nitrogen flux of 7.2 g N m^−2^ d^−1^ using buffer as the catholyte [25]. In the same study, when shifting to MEC mode, the flux of nitrogen increased to 10.3 g N m^−2^ d^−1^, reporting a clear improvement when replacing the catholyte buffer with a NaCl solution (25.5 g N m^−2^ d^−1^). Furthermore, due to the increase in the pH of the catholyte, ammonia stripping was boosted, increasing the nitrogen recovery efficiency in the absorption column to 94.3% [25]. With the same setup, but using digested pig slurry, similar removal rates were reported [2,23,26,27].

In contrast, the low concentration of readily biodegradable organics can hinder current density production and thus ammonia migration, as reported when digested sludge concentrate was used to operate a dual-chambered MEC for nitrogen recovery. The supplementation of primary sludge fermentation liquor (25 vol%) with concentrate achieved a current density of 6.4 A m^−2^, compared with that of <0.15 A m^−2^ obtained using digested sludge alone, with an ammonium removal efficiency of 53% [28]. This improvement was due to the presence of readily biodegradable organic matter in the concentrate. More recently, an excess of 60% of ammonium was recovered from a mixture of anaerobic digester (AD) concentrate and food wastewater (3:1 *v*/*v* ratio) using an MEC. Fertilisers formulated with the recovered ammonia solution were used to grow *Arabidopsis thaliana* as a model plant, confirming that commercial fertilisers can be replaced by the obtained fertiliser supplemented with phosphorus, potassium, and trace elements [29].

A variation in the usual dual-chambered cell equipped with a CEM between the anode and the cathode compartment is the submersible microbial desalination cell developed by Zhang et al. [22]. The aim of this innovative configuration was to recover ammonium and decrease its level in situ in an anaerobic reactor, and it featured an AEM and a CEM outside the anode and cathode chambers, respectively, in contact with the substrate. The authors reported that the external resistance and the initial ammonia concentration influenced ammonia recovery and that ammonia transportation was not negatively affected by the presence of other cations [22].

Dual-chambered MECs with concomitant ammonia recovery and methane production in a biocathode have also been reported, achieving an ammonium flux through the membrane of 14.46 g m^−2^ d^−1^, an average removal efficiency of 30% [30], and an ammonium flux through the membrane of 7 g m^−2^ d^−1^ [31]. However, methane production in the biocathodes of these MECs was 79 L m^−3^ d^−1^ [30] and 92 L m^−3^ d^−1^ [31], respectively.

Recently, the typical configuration of a BES coupled to an external unit for stripping was revisited by adopting a stripping system based on an air diffusion cathode in which air is bubbled. This cathode comprised a high-density polyethylene fibre membrane subjected to a hydrophilic treatment and a layer of unidirectional carbon fibres coated with a Pt-free commercial catalyst. Air was forced through the cathode surface, which provided the oxygen needed for the reduction reaction and allowed for ammonia removal from the catholyte into the acid trap. The BES working with synthetic wastewater recovered 45.3% of the initial nitrogen at a current density of 2.5 A m^−2^. When using real blackwater as the anolyte, the BES achieved a poor performance due to its low content of organic matter and nitrogen [32]. Energy consumption for gas stripping can be also avoided by a passive separation method, facing the cathode directly to the gas phase. This method was applied in a tubular MEC. It was reported that although active aeration achieved a higher ammonia recovery efficiency, the passive separation’s energy consumption was just 1.3 kWh kg_N_^−1^, which was significantly lower than the 2.3 kWh kg_N_^−1^ with active aeration [33].

One of the main drawbacks of traditional BESs coupled to stripping units for ammonia recovery is the low solubility of oxygen, which leads to a lack of electron acceptors. Zhang et al. [34] proposed the introduction of a highly soluble iron complex (tartrate-EDTA-Fe) as an electron mediator (EM) to the catholyte to improve the transport to the cathode of the oxidising equivalent produced at the stripping unit. This allows a current to be produced, inducing ammonium migration thanks to the oxidation of the EM at the cathode. Air then re-oxidises the EM in the stripping column while simultaneously separating the ammonia. The ammonium recovery rate in the EM system was 3.8 times greater than in the non-EM control. This strategy appears to be especially important when upscaling BESs due to the stacking and modularisation design commonly employed for BESs, since the use of an EM will simplify the system design, avoiding the aeration of each cathode compartment and concentrating it only at the stripping unit [34].

MECs have been also combined with forward osmosis (MEC-FO) to recover nutrients, energy, and water, achieving recovery efficiencies of 99.7% and 79.5% for ammonium nitrogen and phosphorus, respectively [35]. An MEC–FO system was developed to recover ammonium from landfill leachate in an MEC, and the recovered ammonium was used as a draw solute in FO for water recovery. The leachate from the landfill was fed into the MEC anode compartment, and its effluent then flowed into the FO feed side. Using external CO_2_, an ammonium bicarbonate solution was prepared with the collected ammonia gas that was then used as the FO draw solution. Thanks to the aeration, ammonia gas was stripped out from the catholyte, thus boosting ammonium removal from the anode chamber [36].

Although laboratory-scale BESs have been shown to be a suitable technology for wastewater treatment, especially when applied to urine ammonia recovery, the upscaling of BESs still presents a challenge. However, steps have recently been taken to understand upscaling effects on BESs. San Martin et al. [37] reported that with an increase in the reactor size from 500 mL to 1000 mL, the ammonia recovery efficiency decreased from 47% to 42% without a significant impact on the current density. However, the main bottleneck was the low biodegradability of the wastes [37]. More recently, a 12 L micro-pilot tubular MEC was developed by Cristiani et al. [38] as a post-treatment for an anaerobic digestion process for simultaneous ammonium recovery and biogas upgrading, which achieved a nitrogen removal efficiency of 36% [38]. The largest dual-chambered CEM BES reported in recent years had a volume of 168 L. Three pilot MECs of this dimension were constructed and installed in different municipal wastewater treatment plants (WWTPs) in Switzerland, generating a renewable chemical base and coextracting abundant species, such as ammonium, from wastewater [39].

Table 1 presents a summary of the main data of the most recent studies using dual-chambered BESs, including their operation mode, type of membrane, anolyte and catholyte, and compartment volume.

### 4.2. Dual-Chambered BESs Combined with Hydrophobic Membrane Modules

The striping and absorption step coupled to BESs in most of the studies referred to in the previous section is highly energy consuming due to continuous air pumping. Hydrophobic membranes (HM) have been tested as an alternative to overcome this issue. Hydrophobic membranes made of gas-permeable materials, such as polyvinylidene fluoride (PVDF), polypropylene (PP), or polytetrafluoroethylene (PTFE), and in the form of tubular, flat, or hollow-fibre membranes, are being coupled to BESs to improve ammonia recovery. Dissolved ammonia gas in a waste stream or in a liquid can pass through the pores of an HM and can be absorbed in an acid solution on the other side, such as sulphuric acid, to form an ammonium salt.

Kuntke et al. [40] reported the recovery of ammonia using an HM module, also called a transmembrane chemisorption (TMCS) module, integrated in the catholyte chamber of a continuously fed MEC. Inside the gas-permeable tubular HM, ammonia was absorbed in sulphuric acid, giving an ammonium sulphate solution. During the 20-day experimental period, the average ammonium removal was 42%, with a maximum removal of 51%. Ammonium was the main contributor to the charge transport through the CEM since on average it represented 70% of the transport efficiency through the CEM, with a maximum of 104% [40]. Different MECs coupled to TMCS units were operated with synthetic and human urine. Each TMCS unit consisted of a PP tubular membrane and was operated in the same way as in the previous study, although in crossflow mode, with the acid on the outside and the catholyte on the inside of the TMCS membrane. The maximum ammonium removal efficiency was 60.9%, which corresponded to a transport rate of 119 gN m^−2^ d^−1^ [42]. This is the highest removal rate reported in the studies included in this review, followed by 86.2 gN m^−2^ d^−1^ reported by Zhang et al. in a submersed MFC [22].

A TMCS module was also connected to an upscaled MEC (volume of 2.5 L for both the anode and cathode chambers) [43]. The authors reported an unstable ammonia transport through the TMCS module due to both the unstable current density production of the MEC and variations in the conditions of the catholyte (i.e., NH_3_/NH_4_+ content and pH), an effect also reported in later research [45]. A high fluctuation in the pH of the catholyte led to unstable ammonia transport through the TMCS membrane. During stable current production, the ammonium transport efficiency through the CEM was 92 ± 25% with a nitrogen recovery of 31 ± 59% [43].

Finally, an electromethanogenic MEC combined with an ammonia recovery system composed of a flat HM was operated with digested pig slurry. The nitrogen transference through the CEM was 8.7 gN m^−2^ d^−1^ on average, achieving a removal efficiency of 31% concomitant with methane production [44].

### 4.3. BESs with Three or More Chambers for Ammonia Recovery

Three-chambered BESs have been tested for ammonia recovery, combining a CEM with an AEM or HM (Table 2). In most cases, ammonia recovery is combined with the production of other value-added products, such as methane.

Xiao et al. [46] proposed a three-chambered MFC to recover not only ammonium from synthetic wastewater, but also nitrate into value-added nitrogenous fertiliser from the anode and cathode chambers, respectively. In the cathode chamber, ferric nitrate was applied as the electron acceptor, which was also used to evaluate the nitrate recovery efficiency. About 47% of the ammonium in the anode chamber and 83% of the nitrate in the cathode chamber were recovered [46].

Zepilli et al. [47] proposed a three-chambered BES configuration to combine ammonium, chemical oxygen demand (COD), and CO_2_ removal in the same device. In this configuration, an intermediate accumulation chamber was placed between the anode and cathode chambers. A CEM and an AEM were placed on the anode and cathode sides of the accumulation chamber, respectively, to recover a concentrated solution of bicarbonate and ammonium ions concomitant with methane production in the cathode compartment. This allows the recovered concentrated solution of ammonium bicarbonate to be used for agricultural fertilisation or in advanced biotechnology applications such as autotrophic microalgae growth [47]. With the same configuration, the ammonium recovery rate and efficiency were improved based on the ammonium loading rate. The ammonium recovery efficiencies obtained in the study are the highest reported to date, achieving a maximum of 75.5%. The highest recovery rate was 38 g_N_ m^−2^ d^−1^) with a 7.4-fold up-concentration of ammonia, reaching 7483 mg L^−1^ in the concentrate [48].

The MEC with three compartments with an intermediate accumulation chamber was improved by adopting a new two-sided cathode configuration, with two cathode compartments separated from the intermediate anodic compartment by an AEM and a CEM and connected in parallel by a titanium wire. The two-sided cathode configuration MEC allowed for the removal of CO_2_ through bioelectromethanogenesis in both cathode chambers, while ammonium migration took place only in the cathode compartment equipped with the CEM. Zeppilli et al. [49] developed this configuration to couple CO_2_ removal from a gas mixture in order to produce CH_4_ alongside the recovery of ammonium nitrogen. Compared with the three-chambered MEC with an intermediate accumulation chamber assessed earlier by the same authors [47], the two-sided cathode configuration performed better in terms of energy consumption, CO_2_ removal, and methane production, maintaining similar anodic performances [49]. The two-sided cathode setup was further modified by replacing the CEM with a PEM. This configuration achieved a 61% ammonia removal efficiency when working in galvanostatic mode (300 mA), compared with 47% when working in potentiostatic mode (+0.2 V vs. standard hydrogen electrode, SHE). The catholyte achieved a concentration of 4060 mg_N_ L^−1^, representing a concentration factor of 4.7 times with respect to the anodic influent [50].

Finally, as in the case of dual-chambered cells, HMs have been assessed as a way of obtaining an ammonia solution free of other cations. Instead of integrating the ammonia recovery system in the catholyte recirculation loop, as described in Section 4.2 and the most commonly used configuration up to now, a three-compartment system MEC can be developed. This last configuration simplifies the operation and construction of the MEC, due to the reduction in the number of vessels, for a future system scale-up. A laboratory-scale MEC with an anode compartment and a cathode compartment, divided by a CEM, and a third compartment placed next to the cathode compartment, separated by a HM, was used for the treatment of organic and nitrogen high-strength wastewater. The flux of ammonium through the CEM was 3.4 g_N_ m^−2^ h^−1^. The ammonia recovery through the HM achieved a flux of 1.5 g_N_ m^−2^ h^−1^, mainly governed by the pH value and the ammonia concentration of the catholyte [45].

BESs aimed at ammonia recovery have evolved in recent years into more complex designs in order to increase the number of products recovered or to reduce energy and reactant consumption. For example, although the use of HM in combination with BESs has represented savings in energy consumption compared with stripping due to the avoidance of aeration, as described above, an acidic solution is still needed to absorb the ammonia that passes through the membrane, contributing to most of the reagent’s cost. An innovative system called a bioelectrochemical membrane-absorbed ammonia (BEMAA) system was designed by Zhang et al. [51], combining a bipolar membrane (BPM) with an HM in order to avoid this acid consumption. A BPM can be applied to the production of a base and an acid in a BES, reducing operational costs with the simultaneous recovery of ammonia from wastewater. This system comprised five chambers and four membranes: The anode chamber was placed next to the acid production chamber, using a BPM; next to the acid production chamber and separated by an AEM was the desalination chamber, where the ammonia-rich substrate was introduced. Ammonium migrated through a CEM towards the cathode chamber, and finally, an absorption chamber was placed on the other side of the cathode compartment, while a piece of HM was installed between the cathode and the absorption chambers. The base generated in the cathode chamber increased the pH value of the catholyte to boost the transformation of ammonium into ammonia, while the acid simultaneously supplied by the BPM in the acid production chamber was transported to the absorption chamber where ammonia was recovered via membrane absorption. The ammonia removal and recovery efficiencies were 97.3% and 68.1%, respectively [51].

Table 2 presents a summary of the main data of the most recent studies, including the number of compartments, operation mode, type of membrane, anolyte and catholyte, and volume of the compartments.

**Table 2:** Recent results on nitrogen recovery using BESs with three or more chambers.

Configuration (Number of Chambers)	Membrane	Anode Volume (mL)	Cathode Volume (mL)	Membrane Surface (cm^2^)	Anolyte	Catholyte	Maximum Current Density (A m^−2^)	N Recovery Rate (g_NH4+-N_ m^−2^ d^−1^)	Energy Demand (kWh kg_N_^−1^)	Combined with	Ref.
MFC (3)	PEM	100	100	n.r.	Synthetic	Fe(NO_3_)_3_ solution	n.r.	n.r.	n.r.	NO^3-^ recovery	[46]
MEC (3)	CEM AEM	35	35	35	Synthetic		4.8	38	n.r.		[48]
MEC (3)	CEM AEM	35	35	35	Reject water		4.0	23	6.1–8.2		[48]
MEC (3)	CEM	600	600	20	Digested pig slurry	NaCl solution	1.4	3.4	n.r.	HM	[45]
MEC (3)	PEM AEM	860	860	289	Synthetic	Synthetic	3.01 ^c^	1.3 ^c^	n.r.	CH_4_ production	[47]
MEC (3)	PEM AEM	860	860	289	Synthetic	Synthetic	5.02 ^c^	2.2 ^c^	n.r.	CH_4_ production	[49]
MEC (3)	CEM AEM	860	860	289	Synthetic	Synthetic	6.82 ^c^	19.7 ^c^	19.66	CH_4_ production	[50]
MEC (3)	CEM AEM	860	860	289	Synthetic	Synthetic	6.92 ^c^	18.2 ^c^	30.62	CH_4_ production	[50]
MEC (3)	CEM AEM	860	860	289	Synthetic	Synthetic	10.4 ^c^	24.8 ^c^	27.46	CH_4_ production	[50]
Tubular MDC (3)	CEM AEM	280	1600	n.r.	Synthetic	Deionised water	n.r.	n.r.	n.r.		[24]
MEC (5)	BPM, AEM, CEM, HM	120	60	16	Synthetic	Synthetic	2.5 ^c^	22	2.91	Acid-base production	[51]

^c ^Calculated from data reported in the paper.

### 4.4. Overview of the Relationship between Nitrogen Removal Rates and Current Density

**Figure 2:**
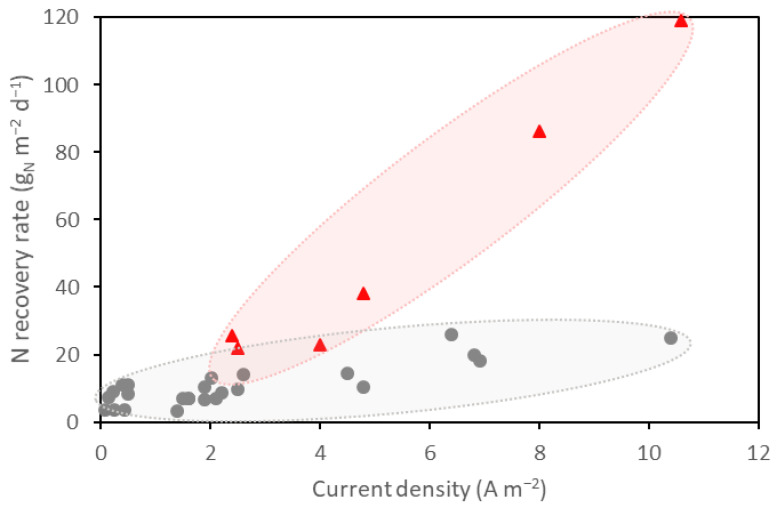
Nitrogen recovery rate plotted against current density for the studies included in this review. Results are clustered into two groups: grey dots for the least efficient N recovery studies, and red triangles for the most efficient N recovery studies.

Data about nitrogen removal rates and current densities reported in the papers included in this review are plotted in Figure 2. The studies have been divided into two clusters. In the first group of results, most of the reported current densities are below 1 A m^−2^, with another important subgroup having current densities in the range of 1–3 A m^−2^, although with little differences in the nitrogen recovery rate. Fewer studies are placed with current densities above 4 A m^−2^, achieving nitrogen recovery rates of between 10 and 26 g_N_ m^−2^ d^−1^. For the second group, due to the differences in substrate, configuration, reactor size, and operation mode between the studies, no determinant factor for a high ammonia removal rate could be identified. The maximum ammonium transport rate of 119 gN m^−2^ d^−1^ [42] was achieved in a study on the load ratio parameter in BESs. The load ratio (calculated as the ratio between the applied current and the nitrogen loading rate) is an important parameter for evaluating the energy input and efficiency of nitrogen removal in processes driven by the current. Operating the system with a higher than optimal load ratio offers no advantage since it consumes more energy and does not increase the removal efficiency [42,52]. Thus, these studies may have had a better load ratio among other possible factors.

## 5. Concurrent Nitrogen and Phosphorus Recovery Using Membranes in BES

Several studies have dealt with the recovery of ammonium, phosphates, and struvite in BESs [53]. The single-chambered cell configuration was tested [54], and the struvite precipitation was reported mainly on the cathode, which reduces the cathode performance and hinders the recovery of the salt [55]. Consequently, two-chambered and multiple-chambered cells have been developed (Table 3).

### 5.1. Dual-Chambered Cells for Nitrogen and Phosphorus Recovery

In a dual-chambered MFC, struvite can be obtained through different approaches, achieving a phosphate recovery of between 83% and 95%. A phosphate-rich substrate can be circulated first in the anode compartment for organic matter removal and then in the cathode compartment. Struvite can precipitate in the cathode compartment when dosing an NH_4_^+^ and Mg solution due to the increase in pH (pH > 8) near the cathode. Operating the MFC at a high COD and a high aeration flow rate in the cathode compartment increased the current density production and phosphorus recovery [56]. Ammonia required for struvite crystallisation can also be obtained by ammonia migration through the CEM [57,58]. With this configuration, the use of an FO membrane instead of a CEM has been reported to decrease BES performance [59]. An upscaled 10 L dual-chambered MFC stack (volume of each unit of 1.2 L) fed with urine for three months used the pH increase in the cathode compartment to recover phosphate and ammoniacal nitrogen as struvite in an external reactor with an efficiency of 90% and 46%, respectively [60]. Since in most P-rich effluents, such as digestate, there is not enough magnesium to induce struvite crystallisation, requiring the addition of an external source, an innovative bioelectrochemical MgO acidolysis method was developed. MgO was used to inhibit the acidification of the anolyte, while Mg^2+^ migrated to the cathode chamber for struvite crystallisation to recover P. However, the presence of ammonium in the anode compartment blocked Mg^2+^ migration, limiting P recovery [61]. Apart from struvite precipitation, nitrogen and phosphorus can be recovered in dual-chambered BESs by other strategies. In dual-chambered cells equipped with a CEM, 80–90% of ammonium recovery from fermented liquor was achieved in the cathode chamber and around 42% of phosphorus in the anode chamber by the formation of polyphosphate granules and polyhydroxyalkanoates (PHAs) in the biomass present in the effluent. Polyphosphate-accumulating organisms removed phosphate and stored it inside cells as polyphosphate, possibly using it for the synthesis of PHAs [62].

Finally, the recovery of nitrogen by dual-chambered CEM-MECs can be coupled with the recovery of water when combined with FO, as described by Zou et al. [35]. First, ammonium migrated from the anode compartment to the cathode compartment. Then, the anolyte was introduced into the feed chamber of the FO for phosphorus concentration and water recovery. After filtering the concentrated feed solution, struvite was recovered. Furthermore, up to 28.7% of the total energy input could be covered by the hydrogen gas generated, giving a specific energy consumption rate of 5.75 kWh kg^−1^ of struvite [35].

### 5.2. Three-Chambered Cells for Nitrogen and Phosphorus Recovery

With the combination of an AEM and a CEM, a three-chambered cell can be constructed for nutrient recovery, as described for ammonium in Section 4.3, with a recovery compartment placed between the anode and cathode compartments. These devices are known as a bioelectroconcentration cells (BEC), a resource recovery microbial fuel cell (RRMFC), or a microbial nutrient recovery cell (MNRC). It has been reported that an MNRC achieved nutrient recovery using wastewater chemical energy. NH_4_^+^ and PO_4_^3−^ were recovered at 2.6 and 1.0 times the amount, respectively, of their initial concentrations in wastewater. From the recovery solution, more than 62% of NH_4_^+^ and 89% of PO_4_^3−^ were crystallised into struvite that could be used as fertiliser [63]. This configuration has been also tested to treat synthetic urine-containing wastewater. In the treatment, the hydrolysis of urea was increased using electrical and microbial processes. The study found that 42% of the total nitrogen and 37% of PO_4_^3−^ were recovered in the middle compartment [64]. A removal efficiency of 59.7% and 42.8% of ammonia and phosphorus, respectively, was reported in a BEC (8.67 kgN m^−3^ day^−1^); however, the concentration of ammonium in the cathode compartment was always higher than the values for the anode effluent. It was reported that approximately 10% of all the nitrogen recovered permeated through the AEM, probably due to a high concentration gradient between the feed and the concentrate, a low permselectivity of the AEM, and the migration of uncharged and/or paired N species such as ammonia. When the concentrate was flash-cooled, relatively large crystals (≥500 μm) were obtained that were identified as NH_4_HCO_3_ [65]. In a recent study, a three-chambered MEC configuration was used to recover phosphate and ammonium from high-strength nitrogen and organic wastewater (digested livestock manure) in order to precipitate struvite externally. The phosphate and ammonium maximum average removal efficiencies were 36% and 20%, respectively [66].

Monetti et al. described some of the barriers to using domestic low-ionic conductivity wastewater in a BEC, since this substrate produces low current densities (<2 A m^−2^), thus achieving a low nutrient recovery. Ammonia was up-concentrated 20.1 times. Potassium, sodium, and other micronutrients essential to plant growth, such as Mg, Ca, Fe, S, and Zn, were also detected in the concentrate. However, in this study, phosphorus precipitated as Mg and Ca salts on the membranes and in the concentrate compartment, and they were not recovered in the liquid concentrate [67]. The low suitability of the substrate was also reported by El Qelish and Mahmoud [68], who designed and operated a self-powered MNRC for the recovery of macronutrients from sludge reject water to obtain a liquid concentrate. The MNRC produced a low current density of 0.98 A m^−3^. The ammonium removal was of 37.1% with a low up-concentration factor of 0.43. When sludge reject water was amended with livestock wastewater (70:30, *v*:*v*), increasing the organic loading rate, the current output increased to a maximum of 14.10 A m^−3^. Ammonium and phosphate up-concentration factors were then 2.19 and 3.41, respectively [68].

BECs have also been combined with FO for nutrient ion recovery from urine. Once NH_4_^+^ and PO_4_^3–^ migrated through the CEM and AEM, respectively, and concentrated in the system’s intermediate compartment, they were further enriched by water extraction from the integrated FO process and were finally recovered as struvite. The ammonia and phosphate removal efficiencies of the system were 83.4% and 88.8%, respectively. Analysis of the operating costs showed that the income, including the struvite recovery and electricity generation, could compensate for the operational costs [69].

Not only have struvite or concentrated nitrogen and phosphorus solutions been recovered from BESs, but microalgae biomass has been as well. A photomicrobial nutrient recovery cell (PNRC) was developed by Jiang et al. [70], which used ion migration for their recovery as microalgae biomass. More than 92% of the total phosphorus, nitrate nitrogen, and ammonium nitrogen were recovered from eutrophic water fed to the internal compartment. Although some of the nitrogen was removed by nitrification and denitrification processes, 69.3% of the ammonia removal was reported to be assimilated by algae, while *Chloroella vulgaris* was responsible for phosphorus removal. Furthermore, *C. vulgaris* supplied oxygen by photosynthesis in the cathode compartment, allowing for higher current densities in the system and boosting the removal and enrichment of nutrient ions [70].

In order to increase removal efficiencies in MNRC, various strategies have been tested in recent years, such as modified carbon and stainless steel materials for anodes. Carbon-based anodes were reported to achieve higher removal efficiencies for ammonia and phosphorus, reaching 98% and 99%, respectively [71].

### 5.3. Membrane Stack Configuration Cells for Nitrogen and Phosphorus Recovery

The membrane stack configuration was developed in order to boost recovery efficiencies [72,73]. Chen et al. presented an in situ nutrient recovery and self-sustaining wastewater purification process developed in a 10 L scale reactor called an advanced microbial nutrient recovery cell (AMNRC). Three recovery compartments and two desalination compartments delimited with AEMs and CEMs were inserted between the anode and cathode chambers. PO_4_^3–^ and NH_4_^+^ were concentrated in the recovery solution using only the energy contained in the wastewater by 490% and 150%, respectively, and were crystallised out of the circulation system as struvite. NaOH was added to the recovery solution to increase the pH to 8.5–9 in the crystallisation process, and MgCl_2_·6H_2_O was supplemented [73]. Another study investigated for the first time how BESs facilitate urea hydrolysis and recover phosphate and nitrogen from diluted source-separated urine. A stacked microbial nutrient recovery cell (SMNRC) was developed with two nutrient recovery compartments and three nutrient removal compartments. The continuous removal of NH_4_^+^ from urine in the anode compartment shifted the reaction balance towards continuous hydrolysis. This allowed for the recovery of nutrients from urine with a low energy consumption and no toxicity concerns, with a recovery ratio of 84.6% and 92.9% for nitrogen and phosphorus, respectively [74]. Following this line of urea hydrolysis induction in BESs and NH_4_^+^ and PO_4_^3−^ recovery, a later study achieved a 73.1% nitrogen removal and an 86.2% phosphorus removal using three recovery compartments and two dilution compartments, each of which was separated by a membrane. Thanks to the alkaline pH value of the recovery solution (8.1–8.3) and the relatively high concentration of nutrient ions, struvite was recovered [75]. More recently, a novel microbial reverse-electrodialysis electrolysis struvite-precipitation cell (MRESC), containing 10 pairs of desalination cells, operated in fed-batch mode, achieved a phosphate removal efficiency of 72% with a struvite crystallisation rate of 7.62 g m^−2^ h^−1^ [76].

A microbial electrochemical nutrient recovery cell (MRC) featuring two desalination compartments was introduced into an anaerobic osmotic membrane bioreactor (AnOMBR), which integrated a FO membrane for physiochemical separation. By using the current density generated in the MRC, phosphate and ammonia were recovered from the bulk solution in the AnOMBR. This integration minimised the diffusion of ammonium through the FO membrane, thus improving the effluent quality and the scaling potential produced by the phosphate. Furthermore, up to 65% of the PO_4_^3−^ and 45% of the NH_4_^+^ were recovered from the influent [77].

## 6. Electricity Consumption of Nutrient Recovery Using Membrane-BES

While most of the recently published studies focusing on ammonia recovery using membrane-BESs include data related to energy consumption estimations (Table 1, Table 2 and Table 3), investigations into the combined recovery of nitrogen and phosphorus are less common.

Generally, the electricity consumption of BESs for nitrogen recovery is compared with the energetic cost of producing fertilisers by the Haber–Bosch process, which accounts for 8.5 kWh kg_N_^−1^. However, this viewpoint does not consider other issues from fertiliser production, such as carbon emissions related to fertiliser formulation and distribution, downstream processing, and transport. However, part of the nitrogen produced will end up in wastewater, which will need an energy-consuming treatment. For this reason, recent research is also taking into account the energy needed for the nitrification/denitrification process in a wastewater treatment plant, which accounts for 12.5 kWh kg_N_^−1^ [38], or the ammonia oxidation process (Anammox^®^) that has a consumption of 4.2 kWh kg_N_^−1^ [78]. Most of the established recovery technologies, such as air stripping, struvite precipitation, electrodialysis, and adsorption, are still not competitive compared with the Haber–Bosch process that benefits from dimensions of scale [79].

The energy consumption reported in the studies included in this review was in the range of 1.17–2.7 kWh kg_N_^−1^, generally considering only the energy supplied for MEC mode operation. The recovery of nitrogen by CEM-MECs combined with FO developed by Zou et al. [35] showed the lowest energy consumption, considering that the hydrogen gas generated could cover up to 28.7% of the total energy input, giving a specific energy consumption rate of 1.17 kWh kg_N_^−1^ recovered [35]. When recirculation or aeration for stripping was included, the values for energy consumption increased to 5.7 kWh kg_N_^−1^ [80] or more. Qin et al. [36] reported 8.5 kWh kg_N_^−1^ in an MEC from which 64.4% of the energy was due to the external power supply, 22.6% was consumed by the recirculation of the anolyte, and the last 13.0% was consumed by aeration in the cathode [36]. The values shown in Table 3 for three-chambered cells are in the range of 20–30 kWh kg_N_^−1^, but all the reported values correspond to methane-producing BESs, increasing the output products of the reactor.

Using an electrochemical cell, i.e., a cell with an abiotic anode independent of organic matter, a high nitrogen transport rate at a low energy input was reported. With a current density of 20 A m^−2^ and an energy demand of 7.25 kWh kg_N_^−1^, the nitrogen removal rate from the influent was 151 gN m^−2^ d^−1^. The current density of 50 A m^−2^ produced the maximum nitrogen transport rate (335 gN m^−2^ d^−1^) with an energy demand of 15.6 kWh kg_N_^−1^ [81]. Although these removal rates are higher than those obtained with BESs due to the higher current densities applied, the energy demand is also higher due to the oxidation of water instead of the oxidation of an organic substrate. However, working in MFC mode reduced the energy consumption to 0.87 kWh kg_N_^−1^ due to electricity only being required for aeration since the MFC produced the energy required for ammonia migration [22]. 

Some authors have evaluated the energy consumption both in galvanostatic and potentiostatic mode operations, since potentiostatic control is the one used in laboratory-scale reactors, especially for anodic biofilm selection, while a galvanostatic control will be a more common electrochemical technique adopted in full-scale processes to maintain the fixed current value. Zeppilli et al. [50] reported that, compared with the potentiostatic operation, the galvanostatic condition increased the current density and, in turn, the recovery of ammonium by 27%. However, due to a decrease in anodic overpotential, the galvanostatic condition increased the energy consumption due to water oxidation in the anode instead of bioelectrochemical COD oxidation [50]. Energy consumption with passive and active aeration has also been compared with reports that an MEC would reduce its electrical consumption with passive aeration to 0.9 kW h m^−3^ of treated water compared with a consumption of 2.1 kW h m^−3^ of treated water with active aeration [33]. After comparing different aeration rates and voltages, Qin et al. concluded that there is a compensation between ammonia recovery and energy consumption since it was reported that a high aeration rate in the cathode compartment improved the ammonia recovery with a low energy consumption of 4.9 kWh kg_N_^−1^, and ammonia recovery was boosted by a low external resistance due to a higher current production, while a low energy consumption was obtained at mild external voltages (e.g., 0.5 V) [80].

Regarding simultaneous nitrogen and phosphorus recovery BESs, most of them are self-sufficient in regard to energy consumption. An SMDC produced net energy (9.5 kWh kg_N_^−1^) from ammonia recovery, assuming a 0.09 kWh kg_N_^−1^ electric energy output, a 0.9 kWh kg_N_^−1^ consumption for aeration, and a 10.3 kWh kg_N_^−1^ energy equivalent for NH_3_ production using the energy consumption for the Haber–Bosch process [22]. Other studies reported that, compared with conventional technologies, the energy consumption of a BEC is not currently competitive when fed with low-conductivity synthetic urine since a high cell voltage is required, resulting in a relatively low current density production and ammonium recovery rate, with a system consumption of 94.4 kWh kg_N_^−1^ recovered [67].

In summary, although the energy consumption is favourable for nitrogen recovery in BES compared with existing technologies, the slow advances in scaling up BESs are hindering their ability to compete with processes already developed on an industrial scale (i.e., Haber–Bosch). An in-depth study of the different parameters involved in electricity consumption in nutrient recovery BESs would be of great importance for upscaling processes since the data supplied from most of the studies included in this review were only based on the voltage applied. 

## 7. Challenges and Future Perspectives

The main challenges of BES application in wastewater treatment have been addressed by different authors, such as upscaling considerations [82] or practical applications [83,84]. Some of the issues are highlighted below.

Although field trials and industrial applications of BESs have been conducted and multiple start-up companies have invested in scaled-up BES demonstrations for the treatment of industrial effluents [85], experiences related to nutrient recovery are still limited. This absence of pilot-scale demonstrations could be attributed to the high cost of membranes and the lack of appropriate electrode modules designed for large-scale applications. Steps are being made towards replacing expensive materials and developing electrodes. The use of graphite-coated stainless steel and carbon felt to fabricate a scalable composite-designed electrode module (total volume of 1 m^3^) has recently been reported with the aim of integrating mainstream wastewater treatments into BESs [86]. The fabrication of separators, electrodes and, structural frames using biocompatible, low-tech, and fully recyclable materials may help expand their applicability, e.g., rubber or starch-based membranes or electrodes of lignocellulosic biomass pyrolysis charcoal (biochar) as electroactive and conductive materials [87].

However, most of the studies reviewed in this paper are still based on synthetic substrates and highlight the difficulty of achieving high recovery efficiencies with real wastewater due to low conductivity or COD that limit the current density. Since nitrogen and phosphorus recovery through membranes is mainly driven by charge neutrality or migration, boosting the current density is essential if recovery efficiencies are to be improved. Multiple-anode modules can significantly enhance current densities [88,89]. Furthermore, real wastewater’s complex ion composition can compete with ammonia and phosphate recovery or facilitate back diffusion [51]. Wastewater’s ionic composition may affect the transport numbers across the CEM [41]; therefore, it is desirable for the membranes to have a greater ion selectivity. The addition of ionophores to CEMs can selectively transport ammonium through the membrane since they provide a binding site with a suitable size for ammonium complexation by bonding hydrogen with the ionophore components [90].

The suitable operation mode in scaled-up systems may differ from that used in laboratory-scale assays and is another important issue that needs to be addressed. Laboratory-scale operation is generally based on poising the anode to a certain potential (chronoamperometric operation mode). For upscaled systems, a chronopotentiometric method, setting the external circuit current density, might be preferred, avoiding fluctuations in the current density and nitrogen flux. However, the potential of the anode may become less suitable for exoelectrogenic bacteria due to changes produced by the applied voltage, the substrate type, and the concentration or the specific microorganisms developed in the biofilm, for example. In turn, lower COD removal efficiencies might be obtained [50].

Nitrogen and phosphate recovery BES configurations vary widely in their products and underlying processes but coincide in their objective of reusing these nutrients for different purposes. The recovered nutrients can be applied directly as fertilisers [29] since it has been reported that chemical fertilisers can be efficiently replaced by the recovered liquid concentrate at a lower cost and with comparable agricultural productivity [68]. However, other possible applications are emerging. Ammonia recovered from wastewater has been proposed as a nitrogen source for microbial protein production, replacing synthetic nitrogen and contributing to the sustainability of the process [91], or for growing bacteria or algae that could be used for the production of biogas or biofuels [92]. In this sense, more research is needed to fully characterise the concentrated solutions recovered from BESs before their reuse in agricultural soils since livestock manure, urine, and other wastewaters may contain significant concentrations of both inorganic (e.g., heavy metals) and organic contaminants (pesticides, herbicides, antibiotics, etc.) as well as pathogenic microbial strains [87].

Finally, the environmental impact of the recovery of nutrients using BESs needs to be evaluated using life cycle assessment (LCA) methodology. Although LCAs of BESs for wastewater treatment have recently been published [93], no specific LCA for nutrient recovery BESs is available. LCAs could help evaluate the environmental benefits of nutrient recovery in BESs concomitant with other products obtained (such as methane or hydrogen) [94] compared with traditional nutrient production technologies (i.e., the Haber–Bosch process and phosphorus mining).

## 8. Conclusions

BESs for ammonia and phosphate recovery by membranes offer several advantages over other technologies. BES operation favours ammonia volatilisation due to the pH increase in the cathode compartment, without requiring an increase in temperature or the addition of chemicals, unlike conventional stripping and absorption processes. The combination of different kinds of membranes in the same BES reactor could lead to systems that are capable of generating the base and acid needed for ammonia stripping and absorption, thus avoiding the consumption of reactants. The suitable selection of the ratio between the applied current and the nitrogen loading rate can lead to a high ammonium recovery (up to 119 gN m^−2^ d^−1^) with energy consumption in the range of 1.2–2.7 kWh kg_N_^−1^ in the future, making it more competitive with the Haber–Bosch process. Membrane-based BESs allow for the simultaneous recovery of ammonia and phosphate, both nutrients of great interest in agricultural systems, with reported removal efficiencies of up to 99%. It would be worthwhile to intensify research into BESs to achieve high recovery efficiencies at the lowest construction cost in order to take the final step towards scaling up and commercialisation. The main drawbacks of scaling up BESs, such as the need for expensive materials to construct the reactors, limitations due to electrode distances in the scaled configurations, or operational conditions, need to be addressed in order to achieve their successful introduction in the market of membrane-based BESs for nutrient recovery.

## Figures and Tables

**Table 1:** Recent results on nitrogen recovery using dual-chambered BESs.

Configuration/Operation Mode	Membrane	Anode Volume (mL)	Cathode Volume (mL)	Membrane Surface (cm^2^)	Anolyte	Catholyte	Maximum Current Density (A m^−2^)	N Recovery Rate (g_NH4+-N_ m^−2^ d^−1^)	Energy Demand (kWh kg_N_^−1^)	Combined with	Ref.
MFC	CEM	500	500	168	Pig slurry	PBS	0.15	7.2	n.r.	Stripping	[25]
MFC	CEM	500	500	168	Pig slurry	NaCl solution	0.07	3.7	n.r.	Stripping	[25]
MFC	CEM	140	80	100	Synthetic	NaCl solution	1.6	6.8	1.6	Stripping	[32]
MFC	CEM	500	500	168	Digested pig slurry	NaCl solution	0.22	8.86	n.r.		[23]
MFC	CEM	500	500	168	Digested pig slurry	NaCl solution	0.4	11.19	n.r.		[27]
MFC submersed	CEM AEM	18	18	9	Synthetic	NaCl solution	8	86.2	0.86 ^b^		[22]
MEC	CEM	500	500	168	Digested pig slurry	NaCl solution	0.43	3.73	n.r.		[26]
MEC	CEM	400	140	38	Reject water	Tap water	6.4	26	5.8		[28]
MEC	CEM	320		400	Diluted urine	n.r.	1.7	n.r.	2.48		[40]
MEC	CEM	200	180	42	Synthetic	Deionised water	1.89	10.2	n.r.		[41]
MEC	CEM	336	336	96	AD concentrate and food WW	Tap water	2.6 ^c^	14 ^c^	2.7		[29]
MEC	PEM	250	250	n.r.	Pig slurry	PBS	0.5	10.9	n.r.		[37]
MEC	PEM	500	500	n.r.	Pig slurry	PBS	0.5	8.3	n.r.		[37]
MEC	CEM	500	500	168	Pig slurry	PBS	4.8 ^c^	10.3	n.r.	Stripping	[25]
MEC	CEM	500	500	168	Pig slurry	NaCl solution	2.4 ^c^	25.5	n.r.	Stripping	[25]
MEC	CEM	500	500	168	Digested pig slurry	NaCl solution	2.01	12.97	n.r.	Stripping	[2]
MEC	CEM	140	80	100	Synthetic	NaCl solution	2.5	9.6	1.6	Stripping	[32]
MEC	CEM	140	80	100	Blackwater	NaCl solution	1.9	6.5	1.6	Stripping	[32]
MEC	CEM	100	180	42.3	Synthetic	Deionised water	1.6	7.1	5.7 ^a^	Stripping	[33]
MEC	CEM	120	180	96	Synthetic	NaCl solution	1.5	6.9	n.r.	Stripping	[34]
MEC	CEM	200	300	100	Synthetic	NaCl solution	10.6	119	1.9	HM	[42]
MEC	CEM	2500	2500	5000	Urine	NaOH solution	1.8	n.r.	1.36	HM	[43]
MEC	CEM	90	90	72	Synthetic	NaCl solution	0.8 ^c^	n.r.	1.17	Water and P recovery, FO	[35]
MEC	CEM	500	500	168	Digested pig slurry	Synthetic	2.2 ^c^	8.64	n.r.	HM and CH_4_ production	[44]
MEC	CEM	500	500	168	Digested pig slurry	Synthetic	4.5	14.46	n.r.	CH_4_ production	[30]
MEC	PEM	860	860	289	Digestate	Synthetic	2.1 ^c^	7 ^c^	n.r.	CH_4_ production	[31]
Tubular MEC	CEM	3140	5060	2355	Synthetic	Synthetic	0.25 ^c^	3.7 ^c^	2.3	CH_4_ production	[38]
Tubular MEC	CEM	500	500	n.r.	Landfill leachate	Deionised water	0.72	n.r.	8.5	Stripping FO	[36]
Tubular MEC	CEM	1159	1300	836 ^c^	Synthetic	Deionised water	0.15 ^c^	n.r.	1.3	Passive NH_3_ separation	[33]

n.r., data not reported; ^a^ including aeration and recirculation energy consumption; ^b^ only for aeration (MFC produced an electric energy output of 0.09 kWh kg_N_); ^c^ calculated from data reported in the paper; PBS, phosphate buffer solution; HM, hydrophobic membrane; PEM, proton-exchange membrane; CEM, cation-exchange membrane; FO, forward osmosis; WW, wastewater.

**Table 3:** Recent results on simultaneous nitrogen and phosphorus recovery using membrane-based BESs.

Configuration	Membrane	Anode Volume (mL)	Cathode Volume (mL)	Membrane Surface (cm^2^)	Substrate	Maximum Current Density (A m^−2^)	Recovered Product	Reference
MFC	PEM	300	300	n.r.	Synthetic	n.r.	Struvite	[56]
MFC	CEM	305	305	n.r.	Synthetic	n.r.	Struvite	[57]
MFC	CEM	45	30	n.r.	Synthetic	1.17	Struvite	[61]
MFC	CEM	28	28	n.r.	Fermented liquor	n.r.	Ammonia solution and P-rich biomass	[62]
MEC	AEM and CEM	600	600	20	Digested pig slurry	0.26	Nutrient solution	[66]
RRMFC	AEM and CEM	28	28	7.1	Synthetic	1.9 ^c^	Nutrient solution	[64]
MNRC	AEM and CEM	110	110	55	Domestic wastewater	0.6 ^c^	Nutrient solution	[63]
MNRC	AEM and CEM	22	22	n.r.	Wastewater	6	Nutrient solution	[71]
MNRC	AEM and CEM	220	220	50	Sludge reject water with livestock wastewater	0.6 ^c^	Nutrient solution	[68]
BEC	AEM and CEM	200	200	100	Synthetic	50	Nutrient solution	[65]
BEC	AEM and CEM	200	200	100	Domestic wastewater	2	Nutrient solution	[67]
PNRC	AEM and CEM	20	20	7.1 ^c^	Synthetic	2	Microalgal biomass	[70]
MEC-FO	CEM and FO	90	90	72	Synthetic		Struvite	[35]
OsBCRS	AEM, CEM and FO	100	100	25	Synthetic	1.1 ^c^	Struvite	[69]
MRESC	AEM-CEM stack	32	35	12	Synthetic	7.9	Struvite	[76]
SMNRC	AEM and CEM stack	21 ^c^	7 ^c^	7.1^c^	Urine	n.r.	Nutrient solution	[74]
MNRC	3 pairs AEM-CEM	315	105	105	Urine	2 ^c^	Struvite	[75]
AMNR	3 pairs AEM-CEM	4000	4000	n.r.	Domestic wastewater	n.r.	Nutrient solution	[73]

^c^ Calculated from data reported in the paper; AMNR, advanced microbial nutrient recovery cell; BEC, bioelectroconcentration cell; MNRC, microbial nutrient recovery cell; MRESC microbial reverse-electrodialysis electrolysis cell; OsBCRS, forward osmosis integrated bioelectroconcentration and recovery system; PNRC, photomicrobial nutrient recovery cell; RRMFC, resource recovery microbial fuel cell; SMNRC, stacked microbial nutrient recovery cell.

## Data Availability

Not applicable.

## Abbreviations

AD	Anaerobic digester
AEM	Anion-exchange membrane
AMNR	Advanced microbial nutrient recovery cell
BEC	Bioelectroconcentration cell
BES	Bioelectrochemical system
BEMAA	Bioelectrochemical membrane-absorbed ammonia system
BPM	Bipolar membrane
CEM	Cation-exchange membrane
COD	Chemical oxygen demand
EM	Electron mediator
FO	Forward osmosis
HM	Hydrophobic membranes
IEM	Ion-exchange membranes
MEC	Microbial electrolysis cell
MFC	Microbial fuel cell
MNRC	Microbial nutrient recovery cell
MRESC	Microbial reverse-electrodialysis electrolysis cell
OsBCRS	Forward osmosis integrated bioelectroconcentration and recovery system
PBS	Phosphate buffer solution
PEM	Proton-exchange membrane
PNRC	Photomicrobial nutrient recovery cell
PP	Polypropylene
PTFE	Polytetrafluoroethylene
PVDF	Polyvinylidene fluoride
RRMFC	Resource recovery microbial fuel cell
SMNRC	Stacked microbial nutrient recovery cell
WW	Wastewater
WWTPs	Wastewater treatment plant
